# Comparison of Diagnostic Accuracy of MRI With and Without Contrast in Diagnosis of Traumatic Spinal Cord Injuries: Erratum

**DOI:** 10.1097/01.md.0000475641.63087.5c

**Published:** 2015-12-07

In the article “Comparison of Diagnostic Accuracy of MRI With and Without Contrast in Diagnosis of Traumatic Spinal Cord Injuries”,^1^ which appeared in Volume 94, Issue 43 of *Medicine*, Tables 3 and 4 were not correctly presented. The article has since been corrected online.
